# A Security Enhanced Encryption Scheme and Evaluation of Its Cryptographic Security

**DOI:** 10.3390/e21070701

**Published:** 2019-07-17

**Authors:** Miodrag J. Mihaljević

**Affiliations:** Mathematical Institute, The Serbian Academy of Sciences and Arts, 11000 Belgrade, Serbia; miodragm@turing.mi.sanu.ac.rs

**Keywords:** encryption, cryptographic security enhancement, erasure error correction, channel with deletion errors, mutual information, channel capacity, the probability of classification error

## Abstract

An approach for security enhancement of a class of encryption schemes is pointed out and its security is analyzed. The approach is based on certain results of coding and information theory regarding communication channels with erasures and deletion errors. In the security enhanced encryption scheme, the wiretapper faces a problem of cryptanalysis after a communication channel with bits deletion and a legitimate party faces a problem of decryption after a channel with bit erasures. This paper proposes the encryption-decryption paradigm for the security enhancement of lightweight block ciphers based on dedicated error-correction coding and a simulator of the deletion channel controlled by the secret key. The security enhancement is analyzed in terms of the related probabilities, equivocation, mutual information and channel capacity. The cryptographic evaluation of the enhanced encryption includes employment of certain recent results regarding the upper-bounds on the capacity of channels with deletion errors. It is shown that the probability of correct classification which determines the cryptographic security depends on the deletion channel capacity, i.e., the equivocation after this channel, and number of codewords in employed error-correction coding scheme. Consequently, assuming that the basic encryption scheme has certain security level, it is shown that the security enhancement factor is a function of the deletion rate and dimension of the vectors subject to error-correction encoding, i.e., dimension of the encryption block.

## 1. Introduction

The main aim of the error-correction codes is overcoming the noise in public communication channels, but there is a long record of results on employment of error-correction coding theory for developing systems for secret communications. These systems belong to one of the following two main categories: the systems without the so called cryptographic keys, as well as the cryptographic keys controlled ones (see [1], for example).

The first coding based technique for secret communication over noisy channels without employment of cryptographic keys have been proposed in [2] where a dedicated coding scheme has been employed which provides secret communication over a public channel under assumption that the wiretapper faces sample collection through the channel with an ϵ higher noise in comparison with the one in the main channel over which communicate the legitimate parties, and a lot of papers have appeared as a follow-up of [2].

Employment of error-correction codes controlled by the cryptographic keys have been addressed in the both two major settings: the secret (symmetric) key setting and the public (asymmetric) key one. The most famous coding based system is McEliece public key encryption system [3] and this proposal has been followed by a number of results on its analysis and alternative proposals. McEleiece public key system is based on difficulty of decoding a random block error correcting code which is NP-complete in the worst case scenario as shown in [4].

Within the secret key cryptographic setting there are the following two major directions of employment error correction coding: (i) developing certain code-based encryption techniques; and (ii) enhancing security of certain lightweight encryption schemes. A number of symmetric key encryption schemes have been reported based on employment of the code-based and noisy channel paradigm. An illustrative and recent example on code-based secret key encryption schemes is the proposal [5] and its cryptanalysis reported in [6] which has shown insecurity of the proposal. The previous example illustrates that design of efficient code based symmetric encryption techniques appears as a tricky issue.

An alternative approach is to employ coding theory in symmetric key crypto-systems for security enhancement of certain lightweight encryption techniques, and goal of this paper is to add some novel results to this approach. Employment of results on error-correction coding and noisy channels for the security enhancement has been reported in a number of papers, and we could identify the following main directions within this approach. One direction is the enhancement employing a model of noisy channel with the additive noise and related coding results. The other direction is employment of the paradigm of the channels with synchronization errors and results on the related coding techniques. Illustrative techniques for security enhancement based on a model of noisy channels with additive errors have been reported in [7,8,9,10,11], and security evaluation of a generic model of these techniques from information-theoretic and computational complexity points of view are reported in [12,13], respectively. The enhancement approach based on the channels with synchronization errors and in particular an encryption approach which involves a communication channel with the errors in the form of *bits insertion* is reported in [14,15].

*Motivation for the work*. According to the above consideration of the topic, security enhancement of lightweight encryption techniques employing results on communication channels with synchronization errors and related coding appears as an interesting issue, and a particular goal could be consideration of the enhancement employing a deletion channel controlled by the secret key. Also, the addressed issue could be considered as a generalization of the shrinking and self-shrinking encryption techniques reported in [16,17], and a way to overcome the reported weaknesses of these techniques (see, [18,19,20], for example).

*Summary of the results*. This paper yields: (i) a proposal of the encryption-decryption scheme for the security enhancement of lightweight block ciphers based on a binary block error-correction coding and a simulator of the deletion channel controlled by the secret key, and (ii) cryptographic security evaluation of the proposed scheme. We suppose that a building component for developing security enhanced scheme is a block encryption algorithm with a known security level (specified by Definition 2), and we consider this algorithm which is the subject of enhancement as the “initial” encryption scheme. Main results of the paper are in Section 2.2 and Section 4.2. Section 2.2 provides a construction for security enhancement of a given encryption scheme employing a suitable block error-correction code for a binary erasure channel which performs mapping ${\left\{0,1\right\}}^{n}\to {\left\{0,1\right\}}^{n^{\prime }}$, $n^{\prime }>n$, and a simulator of a binary channel with the deletions rate *d* controlled by the secret key. The construction is such that the wiretapper faces a problem of cryptanalysis after a communication channel with bits deletion and the legitimate party should only perform the decryption after a channel with bit erasures correctable by the employed error-correction code. The security enhancement is analyzed in terms of the related probabilities, equivocation, mutual information and channel capacity, and it includes employment of certain recent results regarding the upper-bounds on the capacity of channels with deletion errors. Main result of Section 4.2 is Theorem 1 which in a generic way proves the security enhancement showing that the adversary’s probability to win the specified security evaluation game (specified by Definition 1) is reduced for certain factor δ<<1 which upper bound is derived, and it is a decreasing function of the coding parameter *n* and the deletion rate *d*.

*Organization*. The paper is organized as follows. Section 2 proposes a framework for security enhancement based on the secret key controlled simulation of a deletion channel and dedicated error-correction coding. Technical background for the security evaluation is summarized in Section 3. Security evaluation results are given in Section 4, and the final Section 5 provides a concluding discussion.

## 2. A Proposal for the Security Enhanced Encryption

An encryption and decryption algorithm which provide a provably enhanced cryptographic security are proposed in this section. The enhanced security appears as a consequence of the design based on employment of the simulator of a binary noisy channel which appears as the erasure channel at the legitimate party and the deletion one at the wiretapper.

### 2.1. Underlying Ideas

**Figure 1:**
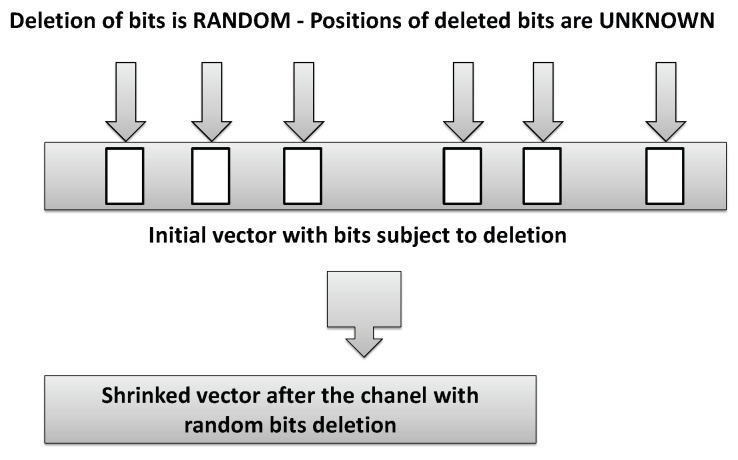
A model of the deletion channel.

The underlying ideas for the design could be summarized as follows. Enhance security of encryption based on information-theoretic and coding results when a wiretapper faces sample collection after a channel with deletions assuming a binary deletion channel with deletion probability *d* which takes input binary string and deletes each bit independently with the probability *d*. A model of the deletion channel is illustrated in Figure 1.

Let a string $Z={\left\{0,1\right\}}^{n}$ denotes an input to a binary deletion channel and let the deletion pattern D is an increasing subsequence of {1,2,…,n} representing the bits that are not deleted. Consequently, $Z_{D}$ denotes the “transformation” of Z after a deletion channel with deletion pattern D.

Note that when the deletion pattern D is known, the deletion channel reduces to the erasure channel and we could consider that $\left(D,Z_{D}\right)$ is the output of erasure channel for given input Z.

**Figure 2:**
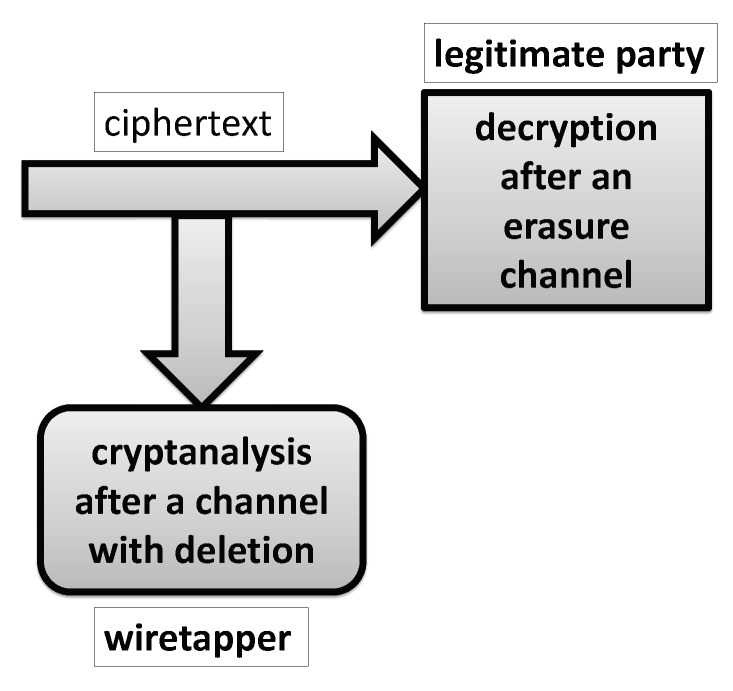
Model of the decryption at a legitimate party versus cryptanalysis at the wiretapper side which faces problem of cryptanalysis after a channel with deletion errors.

The main underlying idea which this paper employs is to enhance cryptographic security of a given encryption scheme in such a way that a legitimate user faces an erasure channel, and a wiretapper faces a deletion channel, i.e., a legitimate party knows the deletion pattern D and a wiretapper does not know this pattern. Assuming that the deleted bits positions are selected in a pseudorandom manner controlled by the secret key and generated by the encryption/decryption algorithm, note that the legitimate party knows D, but the wiretapper who does not know the secret key does not know D and consequently faces a deletion channel instead the erasure one faced by a legitimate party. Accordingly, the corresponding paradigm is displayed in Figure 2.

### 2.2. Framework for Encryption and Decryption

The design proposed in this paper is based on the following building blocks:

- a lightweight block cipher;
- implementation of an error correction code encoding/decoding for binary erasure channel;
- simulation of a deletion channel where the deletion pattern D is generated by the employed block cipher.

It is assumed that encryption and decryption parties share a secret key. As usually, before the session, the both parties (encryption and decryption ones) establish a session key (to be used later on), employing the secret key and the public data.

The encryption and decryption are performed as follows.

- Encryption:
  - a lightweight block cipher generates *n* dimensional binary vector $C^{\prime }=E_{K}\left(M\right)$ where $E_{K}\left(\cdot \right)$ denotes the block cipher encryption according to the secret key K and performs one-to-one mapping ${\left\{0,1\right\}}^{n}\to {\left\{0,1\right\}}^{n}$;
  - an erasure error correction encoding capable to provide correction up to *t* erasure errors generates $n^{\prime \prime }$-bit vector $C^{\prime \prime }$ as the corresponding mapping ${\left\{0,1\right\}}^{n}\to {\left\{0,1\right\}}^{n^{\prime \prime }}$, $n^{\prime \prime }>n$, where *t* is a given parameter, and $n^{\prime \prime }-t>n$;
  - a simulator of a binary channel with random bits deletion performs mapping ${\left\{0,1\right\}}^{n^{\prime \prime }}\;\to \;C\;\in \;{\left\{0,1\right\}}^{n^{\prime \prime }-\ell }$ controlled by a vector X generated by the employed block cipher, ℓ≤t.
- Decryption:
  - an erasure error correction decoding controlled by a vector X generated by the employed block cipher generates *n*-bit vector $C^{\prime }$ by the corresponding mapping ${\left\{0,1\right\}}^{n^{\prime \prime }-\ell }\to {\left\{0,1\right\}}^{n}$, ℓ≤t;
  - a lightweight block cipher generates *n* dimensional binary vector $M=E_{K}^{-1}\left(C^{\prime }\right)$ where $E_{K}^{-1}\left(\cdot \right)$ denotes the block cipher decryption according to the secret key K.

**Figure 3:**
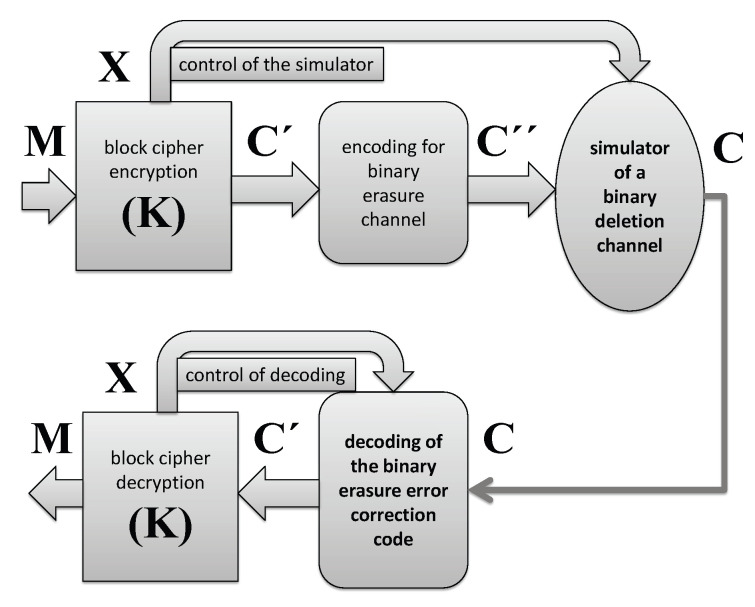
Model of a security enhanced encryption employing a simulator of a noisy channel which appears as a deletion channel from the wiretappers prospective: the upper part shows the transmitter, and the lower part the receiver.

The proposed encryption and decryption framework is displayed in Figure 3.

The objective of this paper is to provide a framework for the security enhancement and show the enhancement gain. Accordingly, consideration of particular instantiations of the framework is out of the scope of this paper. We just point out that a candidate coding scheme could be the polar coding, and that [21] provides an illustrative discussion of polar coding over a binary erasure channel, as well as the decoding complexity after a deletion channel.

Regarding similarity/dissimilarity of the proposed framework and the one reported in [5], note the following. The scheme [5] is based on a suitable block error-correction code and two shift registers which provide that the wiretapper faces a problem of decoding after a channel with flipping, insertion and deletion of the codeword bits. On the other hand, the proposed scheme is based on an (initial) encryption algorithm which has certain security level and a simulator of the deletion channel which in a provable way enhances security of the entire scheme. So, although the block representation of the both schemes has a similarity, they are substantially different because the one reported in [5] is a code-based design of encryption and the one proposed in this paper belongs to a class of the security enhanced encryption employing dedicated coding and simulator of a noisy channel.

## 3. Security Evaluation Background

### 3.1. Notations and Preliminaries

A random variable is denoted by an upper-case letter (e.g., *A*) and its realization is denoted by a lower-case letter (e.g., *a*). The entropy of a random object *A* is denoted by H(A), and the mutual information between two random objects *A* and *B* is denoted by I(A;B). The binary entropy function is denoted by $h\left(p\right)=-p\log_{2}p-\left(1-p\right)\log_{2}\left(1-p\right)$.

The entropy of a random variable *A* is defined as:

(1) $$H\left(A\right):=\sum_{x\in support(A)}Pr\left[A=a\right]\log_{2}\frac{1}{Pr[A=a]},$$

The mutual information I(A;B) between jointly distributed random variables *A* and *B* is defined as follows:

(2) I(A;B):=H(A)−H(A|B)=H(B)−H(B|A)

where conditional entropy is defined as

(3) $$H\left(A|B\right)=\sum_{b\in supp(B)}Pr\left(B=b\right)H\left(A|B=b\right)$$

and

(4) $$H\left(A|B=b\right)=\sum_{a\in supp(A)}Pr\left(A=a|B=b\right)\log_{2}\frac{1}{Pr(A=a|B=b)}$$

Consequently, the conditional mutual information when the third variable *Z* is given is:

(5) I(A,B|Z):=H(A|Z)−H(A|B,Z)=H(B|Z)−H(B|A,Z).

Following [1], the mutual information I(M;C) between the message M and the related sample C, or the uncertainty, i.e., the equivocation H(M|C) are traditionally employed as the main information-theoretic security metric. On the other hand, according to certain recent considerations, the average mutual information $\bar{I}\left(M,C\right)$ should be addressed as a strong information-theoretic security metric, and $\frac{1}{n}\bar{I}\left(M,C\right)$ as a corresponding weak one.

### 3.2. The Probability of Error and The Equivocation after a Noisy Channel

Let *A* and *B* be discrete random variables which correspond to input and output, respectively, of a communication channel. Let the possible realizations of *A* and *B* are $a_{i}$, i=1,2,…,m and $b_{i}$, i=1,2,…,n, respectively, m>n, and let a decision rule on *A* when *B* can be considered as identification of a realization $a_{i}$ when $b_{i}$ is given, and we denote by $P_{err}$ the probability of the identification (classification) error.

Suppose the random variables *A* and *B* represent input and output messages (out of *m* possible messages), and the given conditional entropy H(A|B) represents the average amount of information lost on *A* when *B* is given. According to [22] or [23], for example, we have the following general upper bound on the equivocation:

(6) $$H\left(A|B\right)\leq h\left(P_{err}\right)+P_{err}\log_{2}\left(m-1\right)$$

where h(·)≤1 is the binary entropy function and $P_{err}=1-\mathrm{Pr}\left(A=a_{i}|B=b_{i}\right)$. The above inequality can be rewritten as follows:

(7) $$H\left(A\right)-I\left(A,B\right)\leq h\left(P_{err}\right)+P_{err}\log_{2}\left(m-1\right),$$

and when *A* is such that it has the maximum possible entropy we have:

(8) $$m-I\left(A,B\right)\leq h\left(P_{err}\right)+P_{err}\log_{2}\left(m-1\right),$$

which can be further transformed into:

(9) $$1-\frac{I(A,B)}{m}\leq \frac{1}{m}+\frac{P_{err}}{m}\log_{2}\left(m-1\right).$$

### 3.3. The Capacity of a Deletion Channel

The Shannon capacity of a channel is denoted by Cap and is defined as

(10) Cap:=sup{I(A;B)},

where *A* corresponds the channel input, *B* corresponds to the channel output, and the supremum is over the choice of the distribution of *A*.

As reported in [24], the capacity Cap(d) of a deletion channel with the deletion rate *d* is upperbounded as follows:

(11) $$Cap\left(d\right)=\left(1-d\right)log_{e}\frac{1+\sqrt{5}}{2}$$

for d>1/2, and logarithm is taken to base *e*.

## 4. Security Evaluation of the Enhanced Encryption

### 4.1. Security Notation

We employ a traditional approach for analyzing cryptographic security based on the following two issues: (i) a description of what a “break” of the scheme means, and (ii) a specification of the assumed power of the adversary. A cryptographic scheme is considered as secure one in a computational sense, if for every probabilistic polynomial-time adversary A performing an attack of some specified type, and for every polynomial p(n), there exists an integer *N* such that the probability that A succeeds (where success of the attack is also well-defined) is less than $\frac{1}{p(n)}$ for every n>N. Accordingly, the following two definitions specify a security evaluation scenario and a security statement.

**Definition** **1.** *The Adversarial Indistinguishability Experiment consists of the following steps:*

*1.*  *The adversary A chooses a pair of messages $\left(m_{0};m_{1}\right)$ of the same length n, and passes them on to the encryption system for encrypting.*
*2.*  *A bit b∈{0,1} is chosen uniformly at random, and only one of the two messages $\left(m_{0};m_{1}\right)$, precisely $m_{b}$, is encrypted into ciphertext $\mathrm{Enc}(m_{b})$ and returned to A;*
*3.*  *Upon observing $\mathrm{Enc}(m_{b})$, and without knowledge of b, the adversary A outputs a bit $b_{0}$;*
*4.*  *The experiment output is defined to be 1 if $b_{0}=b$, and 0 otherwise; if the experiment output is 1, denoted shortly as the event (A→1), we say that A has succeeded.*

**Definition** **2.** *An encryption scheme provides indistinguishable encryptions in the presence of an eavesdropper, if for all probabilistic polynomial-time adversaries A*

(12) $$\mathrm{Pr}\left[A\to 1|\mathrm{Enc}\left(m_{b}\right)\right]\leq \frac{1}{2}+\epsilon \;,$$

*where ϵ=negl(n) is a negligibly small function.*

Definitions 1 and 2 are more precisely discussed in [25].

### 4.2. Evaluation of the Security Gain

We consider the encryption/decryption scheme proposed in Section 2.2 which is a security enhanced scheme of certain basic one. Our goal is to estimate the advantage of A in the indistinguishability game specified by Definition 1 when $c\leftarrow \mathrm{Enc}(m_{b})$ where c is a particular realization of C, assuming that the advantage of A is known when $m_{0}$ and $m_{1}$ are two chosen realizations of M and the corresponding realization ${c^{\prime }}_{b}$ of $C^{\prime }$ is given, i.e., the advantage of A is known for the basic (security non-enhanced) scheme.

We assume that in the corresponding statistical model, the considered encryption scheme is such that

(13) I(X,C)=0andI(X,C|M)=0,

i.e., the knowledge of C and M does not leak (provide) any information on X.

**Lemma** **1.** *Let the mapping of m into $c^{\prime }$ be such that $\frac{1}{2}\;+\;\epsilon$ equals the advantage of the adversary A (specified by Definition 2) to win the indistinguishability game (specified by Definition 1). Under these assumptions,*

$$\mathrm{Pr}\left[A\to 1|C=c\right]=\frac{1}{2}+\epsilon \cdot \delta$$

*where*

(14) $$\delta \;\overset{\Delta }{=}\;\mathrm{Pr}\left(C^{\prime \prime }=c_{b}^{\prime \prime }|C=c\right)\;.$$

**Proof.**  For simplicity, it is assumed that $\frac{1}{2}\;+\;\epsilon$ equals the advantage of the adversary A (specified by Definition 2) to win the indistinguishability game. Consequently, let *b* which denotes the index of the selected message be realization of the random variable *B*.

The probability Pr(B=b|C=c) that A wins the game is determined by the following.

(15) $$\begin{array}{cc} \mathrm{Pr}(B=b|C=c) & =\frac{\mathrm{Pr}(B=b,C=c)}{\mathrm{Pr}(C=c)}\\ & =\frac{\sum_{x}\mathrm{Pr}\left(B=b,C=c,C^{\prime \prime }=c^{\prime \prime }\right)}{\mathrm{Pr}(C=c)}\\ & =\frac{\sum_{x}\mathrm{Pr}\left(B=b|C=c,C^{\prime \prime }=c^{\prime \prime }\right)\mathrm{Pr}\left(C=c,C^{\prime \prime }=c^{\prime \prime }\right)}{\mathrm{Pr}(C=c)}\\ & =\frac{\sum_{c^{\prime \prime }}\mathrm{Pr}\left(B=b|C^{\prime \prime }=c^{\prime \prime }\right)\mathrm{Pr}\left(C=c,C^{\prime \prime }=c^{\prime \prime }\right)}{\mathrm{Pr}(C=c)}\;. \end{array}$$

The lemma assumption implies:

(16) $$\mathrm{Pr}\left(B=b|C^{\prime }=c_{b}^{\prime }\right)=\frac{1}{2}+\epsilon \;,$$

where $c_{b}^{\prime }$ corresponds to the selected $m_{b}$, and

(17) $$\mathrm{Pr}\left(B=b|C^{\prime \prime }=c^{\prime \prime }\right)=\frac{1}{2}\;\;\mathrm{for}\;\mathrm{any}\;c^{\prime }\neq c_{b}^{\prime }\;.$$

Note that the encoding mapping $c^{\prime }\to c^{\prime \prime }$ is a deterministic one-to-one mapping and consequently has no impact on the advantage of adversary A, i.e., we have:

(18) $$\mathrm{Pr}\left[A\to 1|C^{\prime \prime }=c^{\prime \prime }\right]=\mathrm{Pr}\left[A\to 1|C^{\prime }=c^{\prime }\right]=\frac{1}{2}+\epsilon \;.$$

Consequently,

Pr(B=b|C=c)=

$$\frac{\mathrm{Pr}\left(B=b|C^{\prime \prime }=c_{b}^{\prime \prime }\right)\mathrm{Pr}\left(C=c,C^{\prime \prime }=c_{b}^{\prime \prime }\right)}{\mathrm{Pr}(C=c)}+$$

$$\frac{\sum_{c^{\prime \prime }:c^{\prime \prime }\neq c_{b}^{\prime \prime }}\mathrm{Pr}\left(B=b|C^{\prime \prime }=c^{\prime \prime }\right)\mathrm{Pr}\left(C=c,C^{\prime \prime }=c^{\prime \prime }\right)}{\mathrm{Pr}(C=c)}\;,$$

Finally, we obtain:

Pr(B=b|C=c)=

$$\frac{\left(\frac{1}{2}+\epsilon \right)\mathrm{Pr}\left(C=c,C^{\prime \prime }=c_{b}^{\prime \prime }\right)-\frac{1}{2}\mathrm{Pr}\left(C=c,C^{\prime \prime }=c_{b}^{\prime \prime }\right)}{\mathrm{Pr}(C=c)}$$

$$+\frac{\frac{1}{2}\sum_{c^{\prime \prime }}\mathrm{Pr}\left(C=c,C^{\prime \prime }=c^{\prime \prime }\right)}{\mathrm{Pr}(C=c)}$$

(19) $$=\frac{1}{2}+\epsilon \cdot \mathrm{Pr}\left(C^{\prime \prime }=c_{b}^{\prime \prime }|C=c\right)\;.$$

QED □

Definition 1 implies that the security of an encryption scheme increases as difference on the adversary A advantage from $\frac{1}{2}$ decreases: The factor δ<1 shows the reduction rate of the advantage, and so we call it the advantage reduction factor.

**Theorem** **1.** *Let the basic encryption mapping ${\left\{0,1\right\}}^{n}\to {\left\{0,1\right\}}^{n}$ of m into $c^{\prime }$, be such that $\frac{1}{2}\;+\;\epsilon$ equals the advantage of the adversary A (specified by Definition 2) to win the indistinguishability game (specified by Definition 1), and the simulated deletion channel has the deletion rate d. Consequently, the advantage of the adversary A, in the security enhanced scheme specified in Section 2.2 is:*

(20) $$\mathrm{Pr}\left[A\to 1|C=c\right]<\frac{1}{2}+\epsilon \cdot \frac{\left(1-d\right)log_{e}\frac{1+\sqrt{5}}{2}+1}{\log_{2}\left(2^{n}-1\right)}\;\;.$$

**Proof.**  According to the (9) we have

(21) $$1-\frac{I(C^{\prime \prime },C)}{n}\leq \frac{1}{n}+\frac{P_{err}}{n}\log_{2}\left(2^{n}-1\right)\;,$$

and taking into account that

(22) $$P_{err}=1-\mathrm{Pr}\left(C^{\prime \prime }=c_{b}^{\prime \prime }|C=c\right)$$

we obtain

(23) $$\frac{1}{n}\mathrm{Pr}\left(C^{\prime \prime }=c_{b}^{\prime \prime }|C=c\right)\log_{2}\left(2^{n}-1\right)\leq -1+\frac{I(C^{\prime \prime },C)}{n}+\frac{1}{n}+\frac{1}{n}\log_{2}\left(2^{n}-1\right)<\frac{I(C^{\prime \prime },C)}{n}+\frac{1}{n}\;,$$

and

(24) $$\mathrm{Pr}\left(C^{\prime \prime }=c_{b}^{\prime \prime }|C=c\right)<\frac{I(C^{\prime \prime },C)+1}{\log_{2}\left(2^{n}-1\right)}\;.$$

Finally, taking into account (10) and (11) we have:

(25) $$\mathrm{Pr}\left(C^{\prime \prime }=c_{b}^{\prime \prime }|C=c\right)<\frac{\left(1-d\right)log_{e}\frac{1+\sqrt{5}}{2}+1}{\log_{2}\left(2^{n}-1\right)}\;.$$

Substitution of (25) into the statement of Lemma 1 yields the proof. QED □

Lemma 1 shows that the encryption mapping m→c enhances the security because the probability that A wins the game becomes closer to $\frac{1}{2}$, which corresponds to random guessing, by the factor δ, and Theorem 1 shows that the upper bound on δ is $\frac{\left(1-d\right)log_{e}\frac{1+\sqrt{5}}{2}+1}{\log_{2}\left(2^{n}-1\right)}<<1$. Accordingly, Table 1 provides a numerical illustration on the upper bound on δ which determines reduction of the advantage of A.

## 5. Concluding Notes

This paper has proposed a framework for security enhancement of certain encryption schemes and its security evaluation. The final security evaluation result given in Theorem 1 also shows the security gain which the security enhanced encryption provides in comparison with the initial one. The lower bound on the security gain is a function of the encryption block size and the deletion rate in the simulated channel with deletion errors. The result given in Theorem 1 is a generic one and it holds for any particular instantiation of the proposed encryption framework.

An interesting future direction is design of particular instantiations of the proposed framework within the given implementation constraints where dedicated basic (initial) encryption, a code for correction of erasure errors and simulator of a channel with deletion errors controlled by the secret key are specified, and complexity of implementation overhead implied by the enhancement is evaluated. Regarding overhead implied by employment of the coding scheme, as an illustration, we point to the polar coding [21] which provides encoding and decoding complexities $O(n^{\prime \prime }\log_{2}n^{\prime \prime })$ assuming that the encoding performs the mapping ${\left\{0,1\right\}}^{n}\to {\left\{0,1\right\}}^{n^{\prime \prime }}$, $n^{\prime \prime }>n$. 

## Figures and Tables

**Table 1:** A numerical illustration of the advantage reduction factor δ upper bound (which shows minimum reduction of the advantage of A) as a function of the encryption scheme parameters *d* and *n*, the deletion rate and encryption block size, respectively.

*d*	Upper Bound on δ for n=64	Upper Bound on δ for n=128
0.55	0.01901	0.00950
0.60	0.01863	0.00931
0.65	0.01825	0.00912
0.70	0.01788	0.00894
0.75	0.01750	0.00875
0.80	0.01712	0.00856
0.85	0.01675	0.00837
0.90	0.01637	0.00819
0.95	0.01600	0.00800
0.99	0.01570	0.00785
